# Acceptability and willingness to pay for telemedicine services in Enugu state, southeast Nigeria

**DOI:** 10.1177/2055207617715524

**Published:** 2017-06-27

**Authors:** Ifeyinwa Arize, Obinna Onwujekwe

**Affiliations:** 1 Department of Health Administration and Management, College of Medicine, University of Nigeria, Enugu-Campus, Enugu State, Nigeria; 2 Health Policy Research Group, Department of Pharmacology and Therapeutics, College of Medicine, University of Nigeria, Enugu-Campus, Enugu, Nigeria

**Keywords:** Telemedicine, healthcare, eHealth, willingness to pay, contingent valuation method, bidding game

## Abstract

**Background:**

This study examines the level of awareness, acceptability and consumers’ willingness to pay (WTP) for telemedicine services using the contingent valuation method (CVM). This work is important as it elicits the value that consumers attach to telemedicine given there is a gap in this knowledge in many sub-Saharan countries such as in Nigeria.

**Methods:**

The study was based on primary data obtained through an interviewer-administered questionnaire of 370 individuals including both males and females from 25 years and over, to collect data on respondents’ awareness of, acceptability of, and WTP for telemedicine, using the bidding game question format. A socioeconomic status (SES) index was created, based on information on household assets, and was used to categorize respondents into SES quartiles. The data were analyzed using a combination of descriptive techniques, logistics and the Tobit regression model (Tobit Type 1) methods.

**Results:**

The study found that majority of the people (58.9%) had no knowledge of telemedicine. However, 48.7% of the respondents were willing to pay for telemedicine. The mean WTP for a telemedicine was US$2.04 for each visit. Tobit regression analysis showed that respondents’ socioeconomic status (SES) was the main statistically significant variable that explained their WTP for telemedicine.

**Conclusion:**

The study has shown that there is a low-level awareness of and WTP for telemedicine services in Enugu State, South East of Nigeria. The finding of a positive relationship between SES and WTP implies that the poor may not be able to pay for telemedicine and may need government subsidies to be able to benefit from such service. Also, government and their partners need to undertake wide scale campaign before the introduction of telemedicine.

## Introduction

Nigeria’s health system is operating in a very complex and dynamic environment. Much of this complexity and change is being driven by the ongoing health sector reform efforts.^1^ Healthcare provision in Nigeria is a joint responsibility of the three tiers of government in the country i.e. the federal, state and local governments. However, private providers of healthcare have a visible role to play in healthcare delivery, with 38% of all registered health facilities being privately owned.^2^

The Nigerian health system is very weak with poor levels of access to services which leads to poor health indices.^3^ Communicable diseases such as malaria and others have been, and still continue to be, a major public health problem in the country.^3–6^ The high prevalence of communicable diseases in Nigeria has now been complicated by the current high incidence and prevalence of non-communicable disease such as hypertension, diabetes and cancer. The overall high disease burden is worsened because of constraints in access to and use of appropriate health services and facilities in Nigeria.^7–9^ Hence, innovative solutions to help improve levels of access and utilisation of healthcare services are required in Nigeria.

In many countries like Nigeria, extending existing healthcare to cover the entire population is therefore a major goal and technology has a strong role to play in this through telemedicine. Information technology (IT) has been identified as a vehicle with the potential to improve the quality and efficiency of healthcare system.^10^ IT has been seen as a possible means of closing the gap of barriers to accessing health care services in the health systems of both developed and developing countries. Telemedicine uses IT to reduce geographic and financial barriers, in order to make access to healthcare services easier. Applications of telemedicine range from the familiar use of the telephone for consultations between patients and clinicians, to the use of radio to link for emergency calls in experimental innovations such as telesurgery.^11^ Evidence of the cost effectiveness of telemedicine has been identified by some studies.^12,13^ Patients can make cost savings, because they do not need to travel long distances to receive their treatment, and their medical diagnosis can also be provided through online consultation or by video conferencing.^11^

Telemedicine can improve the communication and collaboration between physicians and doctors, as they can easily consult each other and share their experiences. Telemedicine has the potential to help in rapidly improving access and utilisation of a wide range of healthcare services and facilities in Nigeria. With support from United Nations Foundations, the Nigerian Ministry of Health developed a National Health ICT Strategic Framework in 2005.^14^ However, telemedicine is still at an early stage in the country despite the fact that it was introduced in the country by January 2008, and that a division on telemedicine exists at the Federal Ministry of Health.^15^ Telemedicine is one of the projects of Nigerian Communication Satellite (NigComSat-1) program, and it is expected that the services will support the improvement of the effectiveness of the Nigerian health system.

Nigeria is a good example of a country in need of telemedicine services with a land mass of 910,771 sq km and a population of about 170 million people and 53% of this population live in the rural areas and healthcare providers are few in number and limited by availability of basic amenities in the country.^16^ Telemedicine can link medical experts with patients in healthcare centers, including in remote areas in which it is difficult to travel and access a specialist doctor.

There is an urgent need to determine the potential demand and acceptability of telemedicine for consumers in Nigeria, to help policy decisions on its scale up, preferences and deployment. It has been noted that the limitations of robust technology and internet utilisation in developing countries makes it harder for people to accept the idea of telemedicine, especially as human beings naturally are averse to change.^17^ The scarcity of resources in low income countries necessitates that health interventions should be acceptable to the general population to ensure use of the services.^18^ Hence, there is need to evaluate acceptability and willingness to pay (WTP) for telemedicine in sub-Saharan African countries including Nigeria.

The assessment of WTP for goods and services, using the contingent valuation method (CVM), is being increasingly used to value healthcare services in developing countries such as Nigeria.^18–22^ Assessment of WTP enables policy makers and health planners to measure the potential demand for products or services. WTP is essentially the maximum amount that an individual is prepared to give up in order to gain utility and satisfaction from the consumption of a particular good or service.^18^

There are very few written studies on WTP for telemedicine, and none in sub-Saharan Africa. One of the available studies established that more than half the patients surveyed at a tertiary care centre were willing to consider using telemedicine.^17^ Another study found that patients were willing to use telemedicine, but more for routine than specialized care, while patients with chronic heart failure were also willing to pay for access to telemedicine to avoid the inconvenience of travelling to a doctor’s office.^13^ Barnighausen, Liu and Sauerborn showed that WTP is affected by the patient’s occupation and the income it provides.^23^

This paper provides new knowledge on the maximum amounts that people are willing to pay for telemedicine, in particular teleconsultation in Nigeria. For this study, teleconsultation includes basic healthcare services, such as a patient consulting a doctor such as a patient consulting a doctor or patient, community health worker and doctor consulting through synchronous or asynchronous either through synchronous or asynchronous media for diagnosis, prevention or treatment. Apart from contributing to our scientific knowledge, this evidence is also useful for government resource allocation decision making, on whether it is worthwhile to invest in telemedicine. The information is also useful for pricing decisions if the government or promoters of telemedicine are interested in cost recovery for the service.

## Method

### Study area

The site for this study was Nsukka senatorial district in Enugu State, Nigeria. Nsukka is located in the north of Enugu State and is both a rural and an urban area with the University of Nigeria community, with the town and its surroundings making up the urban area. The people are mostly farmers, while public service in the university is the second most common occupation in the senatorial district. Health facilities in the area include a mostly primary healthcare center in each town, a district hospital and a missionary owned hospital.

### Data collection

Data on the respondents’ awareness of, acceptability of, and WTP for telemedicine was collected from a random sample of 370 individuals, including both males and females of 25 years and over who were either resident in and/or pursuing livelihood activities in the Nsukka senatorial district. The sample size was calculated using a power of 80% and confidence limit of 95% to yield a minimum sample size of 370. A pre-tested, interviewer-administered questionnaire was the data collection tool.

Data was collected on the sociodemographic and socioeconomic status of the respondents. However, data on income was not collected, because such information is not reliable in the study area asmost people are employed in the informal sector.

In addition, data was collected on the respondents’ awareness of and acceptability of telemedicine, to research their preferences before eliciting their maximum WTP amounts for the service. The maximum amounts that people were willing to pay were elicited using the bidding game (BG) question format. The BG has been shown to be valid WTP elicitation question format in Nigeria.^21,24^ The respondents were presented with the hypothetical scenario of telemedicine. In the BG iteration, they were then asked what the maximum amount they would be willing to pay for telemedicine services, per episode of the service. A uniform starting point of ₦500 (US$3) was used. The BG iteration is shown in Box 1. The final response that was indicated was the respondents’ maximum WTP.

**Box 1.** Bidding Game Iteration.

1. The price of a monthly Teleconsultation access is ₦ 500, are you willing to pay?
0 = YES[ ] 1 = NO[ ] (If answer is ‘yes’ go to No 2 if answer is ‘No’ go to No 3)
2. What if the price is ₦600, will you be willing to pay?
0 = YES[ ] 1 = NO[ ] No matter the answer, go to Question 4
3. What if the price is ₦400, will you be willing to pay?
0 = YES[ ] 1 = NO[ ] No matter the answer go to Question 4
4. What if, due to inflation or other factors, the price increases? What is the maximum price you will be willing to pay per month for yourself for telemedicine services bearing in mind your average monthly income and other expenses.[ ] Naira (₦)

### Socioeconomic status

A socioeconomic status (SES) index was created based on information on household assets such as television, motorcycle, radio, etc. as well as per capita weekly food expenditure. The SES index was used to divide the households into different quartiles. It was then used to examine the relationship between WTP and socioeconomic status.

### Data analysis

The data were analyzed using a combination of descriptive techniques, logistics and Tobit regression model (Tobit Type 1) methods. Tabulations were used to analyze the data on the sociodemographic and socioeconomic characteristics of respondents; their level of acceptability of and descriptive statistics on their WTP for telemedicine services. The summary statistics included mean and standard deviations.

In examining the construct validity of the results, logistic regression was first used to explore the explanatory factors that people gave for stating either yes or no to the starting point WTP bid. This explained why they were either willing or not willing to pay for telemedicine. Then, the Tobit regression model (which is a form of censored regression model) was used for determining the validity of the maximum WTP amounts. Tobit regression is appropriate as it has been argued that the appropriate model for limited dependent variables depends on the underlying assumptions behind the zero responses.^25^ If zero responses reflect genuine WTP values of zero, the Tobit model is appropriate for the estimation. In logistic regression, the coefficient (B) values determine the probability of a variable identifying a respondent falling into a particular category (not willing to pay/willing to pay). A negative or positive value shows the direction of the relationship, that is, factors that will increase the likelihood of a yes answer or a no answer. From the coding of the dependent variable in which (0 = no, or ‘not willing to pay’, and 1 = yes, or ‘willing to pay’), negative B values indicate that an increase in the dependent variable will result in a decreased probability of the study case recording a core of 1 in the dependent variable (WTP).

## Results

### Demographic information

The results show that out of the 370 respondents, 56% were males and their mean age was 43 years. Most of the respondents had varying degree of educational levels, with the predominant groups having first degree education (33.5%) and senior secondary (21.6%).

### Awareness of telemedicine services

The results show that 65.1% of the respondents had never heard of telemedicine. However, there was a significant difference in level of awareness depending on geographical location, educational level and occupation. Most of the respondents that had heard of telemedicine were either health personnel or those in the information technology field. Information on telemedicine was obtained via international television channel, journals or magazine.

### Acceptability of telemedicine services

It was found that 62.9% of the respondents agreed that they would benefit from using telemedicine, while 51.1% agreed that they would be willing to use the service. 45.1% of the respondents were willing to use telemedicine for treatment of common illnesses such as malaria or the common cold, but were not willing to use it for a serious illness even if it was routine checkup of such serious illnesses.

### Willingness to pay for telemedicine services

Table 1 shows that the mean WTP value was ₦407 (US$2.03) per visit as the final response.

**Table 1.** Willingness to pay for telemedicine.

WTP for telemedicine	Response
Willing to Pay *n* (%)	181(48.9%)
Mean WTP (SD)	407(263)
Median	250

WTP: willingness to pay.

### Logistic regression analysis result

Table 2 shows the logistic regression analysis of the relationship between the dependent variable (WTP) and independent variables. It shows that there was a positive and statistically significant relationship between the socioeconomic status of the respondents and their WTP for telemedicine. No other factor was statistically significantly related to the decision to answer either yes or no to WTP for telemedicine.

**Table 2.** Logistic regression analysis of overall model.

Model	WTP	
	B	SE
(Constant)	.433	.197
Age of respondent	−.002	.002
Gender of respondent	.031	.037
Marital status of respondent	.040	.039
Occupation of respondent	−.002	.019
Educational level of respondent	−.033	.025
Geographic location	.010	.060
Economic Status	.164	.039
Whether respondent will benefit	.064	.034
Whether respondent will use	.012	.041
Respondent prefers to see a doctor	−.038	.027

B: Beta coefficient; WTP: willingness to pay.

### Tobit regression analysis result

The Tobit regression analysis (Table 3) reveals that for any unit increase in the socioeconomic status of the respondent, the log odds of WTP for telemedicine will increase by 271. However, no other factor predicted the maximum WTP amount.

**Table 3.** Tobit regression analysis.

Independent Variables	WTP
	Coefficient (SE)
Age of respondent	−.044 1.18
Gender	25.23 21.74
Marital status	15.03 22.91
Occupation	−11.84 11.08
Level of Education	−4.67 14.56
Whether respondent will benefit	22.65 20.05
Whether respondent will use	20.64 24.41
Respondent prefers to see doctor	−6.94 15.49
Geographic location	8.20 35.24
Socioeconomic Status	271.51 23.04***
Number of observations	370 370
F statistics	−359.55 115.89**
Adjusted R^2^	0.06 0.05

** significant variable. ***p < 0.01

F: f-distribution; R: correlation coefficient; WTP: willingness to pay.

## Discussion

The findings show that there is a low level of awareness of telemedicine services among the respondents, which is similar to findings by Taylor and colleagues.^26^ This low level of awareness could be attributed to the fact that the technology is still in its early stages in Nigeria. The findings on the difference of awareness depending on where the person lived, their level of education and occupation provide a basis for determining the areas to target awareness creation for the service. For instance, as awareness was higher among those in the urban areas compared with those in the rural areas, more intensive awareness campaigns are needed in the rural areas.

The mixed findings on acceptability show it is possible that people are potentially willing to demand new innovations in access to healthcare services. This study is in line with some evidence from previous studies which suggest considerable patient acceptance of telemedicine in some settings.^26^ There are issues peculiar to Nigeria that make citizens wary of the usefulness of the technology. For example, a lack of a constant power supply, which most of the respondents pointed out, is a reason why most people are concerned about reliability and effectiveness in using telemedicine.

The findings from the personal interviews indicated that the majority of respondents preferred the option of using telemedicine when it offered them access to healthcare that reduced their hospital visit by even 2 hours. This suggests that the majority of respondents will choose telemedicine if the access fee is low, since it will give them faster access. However, some respondents were very emphatic about their preference for the traditional face-to-face doctor–patient interaction. They felt that telemedicine will never be the same as face-to-face interaction, and that it will be akin to talking to a robot about their health challenges.

It was not surprising that majority of the respondents were not willing to pay for telemedicine given the low level of awareness of the technology amongst the citizens. In addition, the acceptability and use of the service is generally low, especially where socioeconomic status been a determining factor of WTP.

The finding from this study that WTP was dependent on socioeconomic status is in line with the study, that economic status is a factor affecting WTP.^21,^ ^27^ It has also confirmed the study by other authors, that a positive increase in economic status will influence technology acceptance and WTP.^17,^ ^28^

The finding of a positive relationship between SES and WTP implies that the poor may not be able to pay for telemedicine, and may need a government subsidy to be able to benefit from such services. That is, those that will use the service will be those that can afford the access fee. This implies that telemedicine may end up being available for only the better-off socioeconomic groups who are willing to pay the high access fee. This ‘exclusion effect’ has been noted in many health intervention services.^29,^ ^30^ It may also mean that if people cannot pay the access fee at a level necessary for the service, then the service may have to be adjusted in line with what people are willing to pay. This might result in poor or low quality service.^30,^ ^31^

Further studies should be conducted to research other financing mechanisms to enable the adoption of telemedicine, since consumer WTP is low and the government might not be able to subsidize the service. Patients’ socioeconomic status, and the affordability of the service are vital factors during any decision making process regarding WTP for telemedicine. It is important to note that a limitation of the study was that the sample in the study was limited to one senatorial district in southeast Nigeria. Secondly, very few studies have assessed cost effectiveness of telemedicine.

In conclusion, a potential market exists for telemedicine in Nigeria if an awareness of the service is created. This study supports the WHO report which shows that African, eastern Mediterranean and Southeast Asian regions have the highest projected growth in the use of telemedicine, and will require extra support in the development of telemedicine policies and strategies in the near future.^32^
